# Ultrafast T2-weighted MR imaging of the urinary bladder using deep learning-accelerated HASTE at 3 Tesla

**DOI:** 10.1186/s12880-025-01810-1

**Published:** 2025-07-15

**Authors:** Li Yan, Qinxuan Tan, David Kohnert, Marcel Dominik Nickel, Elisabeth Weiland, Felix Kubicka, Paul Jahnke, Dominik Geisel, Moritz Wagner, Thula Walter-Rittel

**Affiliations:** 1 Department of Radiology, Charité - Universitätsmedizin Berlin, Corporate Member of Freie Universität Berlin, Humboldt-Universität zu Berlin, Berlin Institute of Health, Berlin, Germany; 2 MR Application Predevelopment, Siemens Healthcare GmbH, Erlangen, Germany

**Keywords:** Magnetic resonance imaging, Deep learning, HASTE, Turbo spin echo sequence, Bladder imaging

## Abstract

**Objective:**

This prospective study aimed to assess the feasibility of a half-Fourier single-shot turbo spin echo sequence (HASTE) with deep learning (DL) reconstruction for ultrafast imaging of the bladder with reduced susceptibility to motion artifacts.

**Methods:**

50 patients underwent pelvic T2w imaging at 3 Tesla using the following MR sequences in sagittal orientation without antiperistaltic premedication: T2-TSE (time of acquisition [TA]: 2.03–4.00 min), standard HASTE (TA: 0.65–1.10 min), and DL-HASTE (TA: 0.25–0.47 min), with a slice thickness of 3 mm and a varying number of slices (25–45). Three radiologists evaluated the image quality of the three sequences quantitatively and qualitatively.

**Results:**

Overall image quality of DL-HASTE (average score: 5) was superior to HASTE and T2-TSE (*p* < .001). DL-HASTE provided the clearest bladder wall delineation, especially in the apical part of the bladder (*p* < .001). SNR (36.3 ± 6.3) and CNR (50.3 ± 19.7) were the highest on DL-HASTE, followed by T2-TSE (33.1 ± 6.3 and 44.3 ± 21.0, respectively; *p* < .05) and HASTE (21.7 ± 5.4 and 35.8 ± 17.5, respectively; *p* < .01). A limitation of DL-HASTE and HASTE was the susceptibility to urine flow artifact within the bladder, which was absent or only minimal on T2-TSE. Diagnostic confidence in assessment of the bladder was highest with the combination of DL-HASTE and T2-TSE (*p* < .05).

**Conclusion:**

DL-HASTE allows for ultrafast imaging of the bladder with high image quality and is a promising addition to T2-TSE.

## Background

Non-invasive imaging is commonly used to evaluate various urinary bladder conditions in clinical practice. MRI is particularly well suited for this purpose due to its high soft tissue contrast [1, 2]. To achieve high spatial resolution T2-weighted (T2w) imaging, turbo-spin-echo (TSE) sequences are typically performed with a slice thickness of 3–4 mm and a small FOV [3, 4]. However, T2-TSE has some limitations including long acquisition times, susceptibility to breathing artifacts as well as bowel and urinary bladder motion artifacts [5, 6, 7], especially when antiperistaltic agents are restricted [8, 9].

Compared to T2-TSE imaging, half-Fourier acquisition single-shot turbo spin echo (HASTE) sequence enabled significant time reduction and has been widely used in various clinical settings [10, 11]. However, the faster sampling of k-space in the HASTE sequence can lead to blurriness and low SNR, which may result in insufficient detailed information for accurate diagnosis [12, 13].

DL reconstruction is a cutting-edge technology that has been applied to various MR sequences, allowing for great reduction in acquisition time (TA) and improved SNR [14]. The application of DL-accelerated T2-TSE sequence in pelvic MRI already showed significant improvement in image quality [15, 16], while combining DL reconstruction with the HASTE sequence permits even shorter acquisition time. Recent investigations in upper abdominal imaging showed that DL-HASTE allowed single-breath-hold acquisitions with high image quality and preserved realistic texture compared to navigator-triggered T2-TSE [17, 18, 19]. However, to the best of our knowledge, the use of DL-HASTE in bladder imaging has not been evaluated yet.

Based on the hypothesis that DL-HASTE could also benefit bladder imaging, we aimed to compare DL-HASTE with standard HASTE and T2-TSE, and hereby evaluate the feasibility of ultrafast MR imaging of the bladder using DL-HASTE at 3 Tesla.

## Methods

### Study design

The institutional ethics review board approved this exploratory, prospective study. Written and informed consent was obtained from all participants. This study was conducted in accordance with the ethical standards as laid down in the 1964 Declaration of Helsinki and its’ later amendments.

**Fig. 1:**
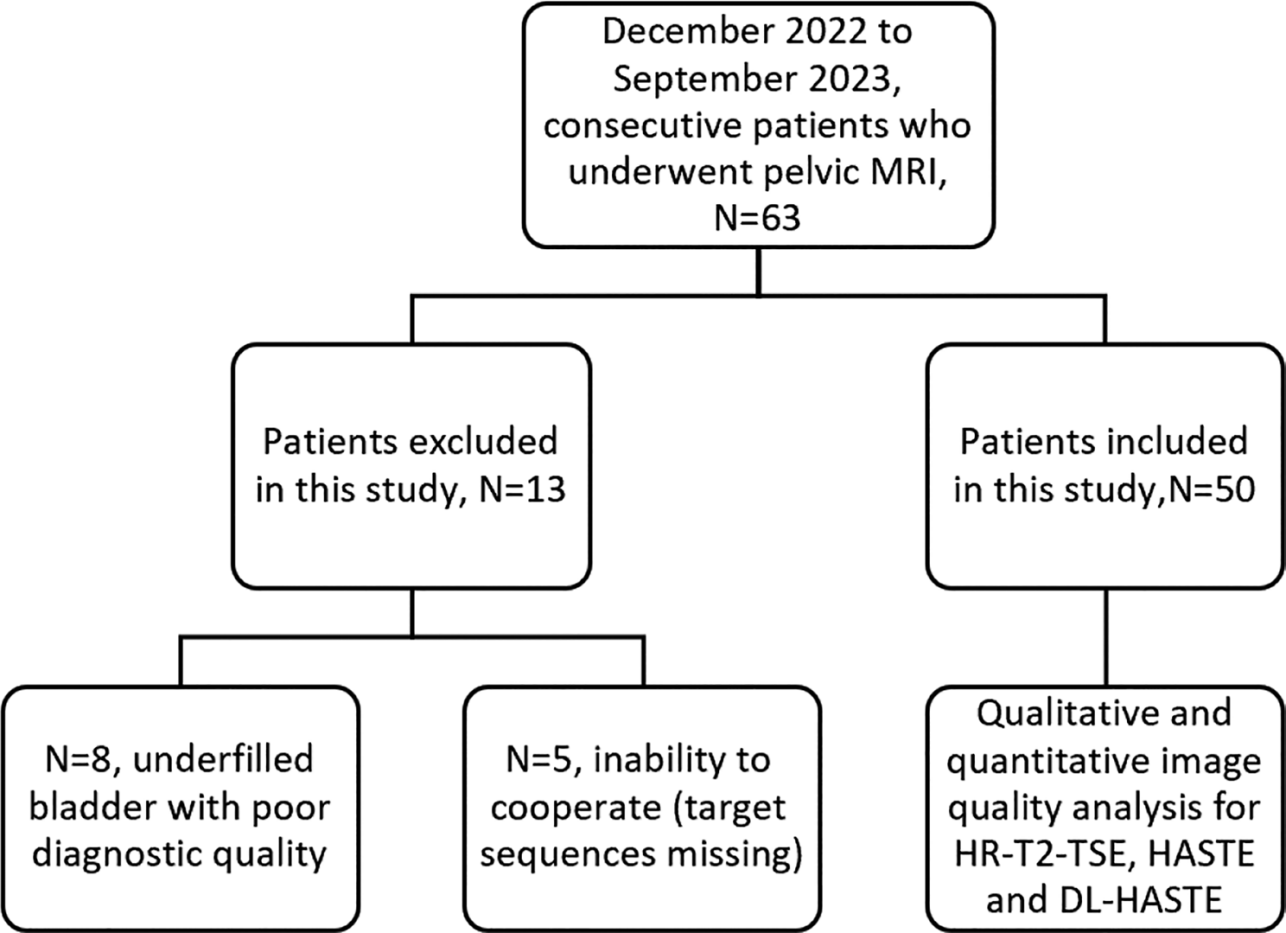
Flowchart of patient inclusion process. HR-T2-TSE, high spatial resolution T2 turbo-spin-echo; HASTE, half-Fourier single-shot turbo spin echo sequence; DL-HASTE, deep learning half-Fourier single-shot turbo spin echo sequence

This study prospectively included 50 adult patients who underwent pelvis MRI on a scanner equipped with a DL reconstruction algorithm, without the administration of butylscopolamine. The DL reconstruction algorithm used in this study is a research prototype developed in collaboration with Siemens Healthineers and is not currently commercially available. Clinical indication: diseases of low urinary tract, low urinary tract symptoms due to compression or invasion by a pelvic tumor outside the bladder as well as irritation from pelvic inflammatory disease. Exclusion criteria: (1) the patient’s inability to cooperate, resulting in absence of the target sequences; (2) underfilled bladder with thickened and irregular bladder walls that might obscure luminal structures with a volume well below the recommended 300 ml, determined in consensus by two experienced radiologists during initial image review, to ensure diagnostic interpretability. The final patient group consisted of 27 males and 23 females, with a mean age of 57 ± 16 (range, 23–87 years; Table 1; Fig. 1).

**Table 1:** Patients’ demographics

Variable			
Total (male/female), n		50 (27/23)	
Age, mean ± SD (range), y		Total: 57 ± 16 (23–87)	
		Male: 58 ± 15 (29–87)	
		Female: 55 ± 16 (23–73)	
Diagnosis, n		Normal bladder, 27	
		Bladder cancer, 12	
		Bladder diverticula, 4	
		Bladder inflammation, 5	
		Bladder fistula, 1	
		Bladder infiltration (rectum cancer), 1	
Indication of MRI, n		Primary staging, 17	
		Follow up, 13	
		Other, 20	

### MRI acquisitions

All examinations were performed on a clinical 3T scanner (MAGNETOM Vida; Siemens Healthineers, Erlangen, Germany) with patients in supine position using an 18-channel spine array. The protocol of this study consisted of the following sequences: (1) sagittal high spatial resolution T2-TSE; (2) sagittal standard HASTE; (3) sagittal DL-HASTE. Detailed information about acquisition parameters is given in Table 2. The DL reconstruction for the HASTE sequences is a research application, as described previously [17, 20]. The DL-HASTE acquires k-space data using a regular sampling as known from parallel imaging with a separate acquisition of the calibration data to estimate coil sensitivity maps. To reduce crosstalk in acquisitions, the slice increment between consecutively acquired lines is increased to 4. Additionally, a variable flip angle evolution is supported for the refocusing pulses in the echo train [17, 21]. The DL reconstruction is based on a variational network [14]. Compared to versions used in earlier works [20, 22, 23, 24], the network architecture was modified. In particular the regularization component was altered to a hierarchical design as first explored in the context of prostate imaging [14]. The hierarchical design of the altered regularization component aimed to enhance edge preservation and reduce noise in high-resolution images.

**Table 2:** Acquisition parameters

Parameters	T2-TSE	HASTE	DL-HASTE
Orientation	Sagittal	Sagittal	Sagittal
TA, min	4.00	1.10	0.47
TE, ms	106–108	96	95–98
TR, ms	> 3000	1100	500
FA, degree	Fixed FA 160	Fixed FA 100–160	Variable FA 130, 90, 110,130
*Matrix	384 × 311	384 × 311	384 × 311
Slice thickness, mm	3	3	3
*Number of Slices	45	45	45
*FOV, mm^2^	250 × 250	300 × 300	300 × 300

TA, time of acquisition; TE, echo time; TR, repetition time; FA, flip angle. *: Default values

### Qualitative MRI evaluation

Sagittal pelvic T2w-images were anonymized and independently assessed in a random order by three radiologists with 15, 6 and 5 years of experience in MR imaging, respectively. The images of T2-TSE, standard HASTE, and DL-HASTE were simultaneously presented to the readers. All readers used a 5-point Likert scale to evaluate the following parameters (the regarding criteria are listed in Table 3)

- Overall image quality: taking into account image noise, homogeneity of signal intensity, depiction of bladder wall and its surrounding structures, and motion artifacts.
- Bladder wall/ureterovesical junction delineation: taking into account sharpness, contrast, motion artifacts, depiction of bladder wall and its surrounding structures.
- Breathing artifact: regular spaced ghosting is mainly caused by the anterior abdominal wall.
- Urine flow artifact: heterogeneous signal intensity within bladder lumen.
- Bowel motion artifact: irregular “blurring” ghosting artifacts around bowel wall due to peristalsis.
- Diagnostic confidence: evaluating both bladder lumen and bladder wall based on T2-TSE, standard HASTE, DL-HASTE, and combination of TSE with HASTE or DL-HASTE, respectively.

As bladder image quality can vary by region due to different motion artifacts and bladder filling conditions, readers qualitatively assessed the bladder in anatomical subdivisions such as the bladder dome, base, anterior wall, and posterior wall.

**Table 3:** Scoring criteria for qualitative image evaluation

Score	Overall image quality (IQ)	Bladder wall delineation	Ureterovesical junction delineation	Artifacts*	Diagnostic confidence
5	Excellent	Well-displayed	Well-displayed	Absent	High certainty
4	Good	Minimal blurred	Minimal blurred	Minor	Sufficient certainty
3	Moderate	Moderate blurred	Moderate blurred	Moderate	Low certainty
2	Substantially impaired	Substantially blurred	Substantially blurred	Substantial impact on diagnosis	Uncertain
1	Non-diagnostic	Severely blurred	Severely blurred	Non-diagnostic	Non-diagnostic

^*^: applies to breathing artifact, bowel motion artifact and urine flow artifact

### Quantitative MRI evaluation

Quantitative image evaluation of sequences (T2-TSE, standard HASTE, DL-HASTE) was performed by two readers in consensus. For each sequence, four circular ROIs were placed in the following locations: the fatty zone in front of the bladder (ROI 1), the fatty zone above the bladder (ROI 2), the fatty zone under the bladder (ROI 3), and the muscular area of lower abdominal wall (ROI 4) or pelvic floor if the lower abdominal wall was obscured by the anterior saturation band on T2-TSE.The manually drawn ROIs were placed in the same slice for each sequence in every case, whenever possible. The size of each ROI was between 200 mm^2^ to 250 mm^2^.

Image noise was denoted by the SD value. For each of the image sets, the fat SNR, the CNR between fat and muscle were calculated using the following formulae respectively:

Fat-SNR = SI_fat_ / SD_fat_.

Muscle-fat CNR = (SI_fat_ - SI_muscle_) / SD_muscle_.

The signal intensity (SI_fat_) and corresponding SD_fat_ were calculated as the mean value of ROI 1–3.

### Statistical analysis

All statistical analyses were performed using SPSS version 27 (IBM Corp, Armonk, NY). Continuous variables were described using the mean and standard deviation, and ordinal variables were described using the median and IQR. Kolmogorov Smirnov test was applied to assess the normality of variables. Using Wilcoxon signed-rank test to analyze reading scores of T2-TSE, HASTE and DL-HASTE sequences. Continuous variables were compared using paired samples t-test for normal distribution or Wilcoxon test for skewed distribution, P values less than 0.05 were considered to indicate a significant difference. The inter-observer agreement was evaluated with intraclass correlation coefficient (ICC). Based on the 95% confident interval (CI) of the ICC estimate, the value was considered as follows: 0-0.50 = poor reliability, 0.51–0.75 = moderate reliability, 0.76–0.90 = good reliability, 0.91-1.00 = excellent reliability.

## Results

**Fig. 2:**
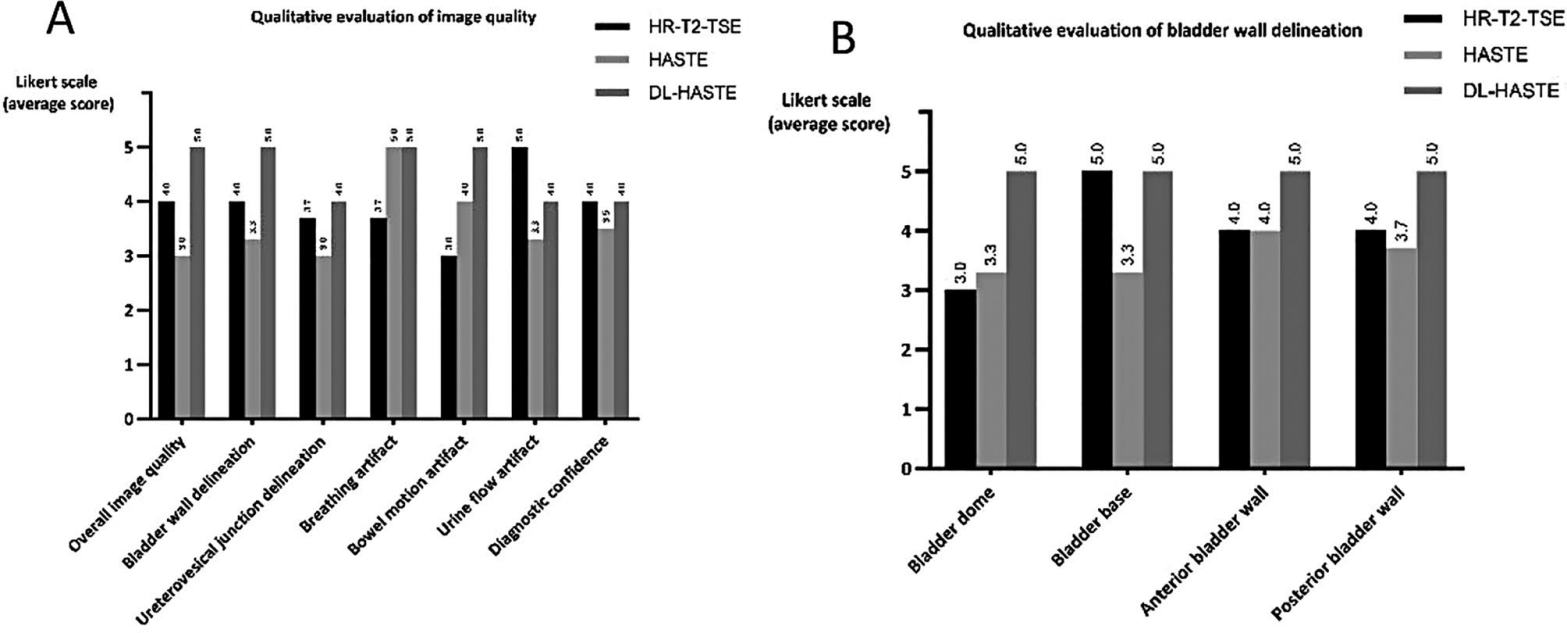
Averaged ratings across three readers for qualitative evaluation of image quality (**A**) and for qualitative evaluation of bladder wall delineation

The acquisition time for T2-TSE was 2.03 to 4.00 min, while for standard HASTE it was 0.65 to 1.10 min. DL-HASTE were technically successfully acquired in all patients, with the shortest scanning time ranging between 0.25- and 0.47-min. Inter-observer agreement for qualitative parameters was moderate to good with ICC values ranging from 0.645 to 0.853. In the following, the average scores from three readers are described in the text, Fig. 2 is the corresponding histogram. Detailed results of all readers are shown in Tables 4 and 5. Statistical differences among T2-TSE, standard HASTE and DL-HASTE in terms of qualitative evaluation are presented in Table 6, based on all original ratings from three readers.

**Table 4:** Qualitative evaluation of image quality

	T2-TSE			ICC	HASTE			ICC	DL-HASTE			ICC
	Reader 1, Median (IQR)	Reader 2, Median (IQR)	Reader 3, Median (IQR)		Reader 1, Median (IQR)	Reader 2, Median (IQR)	Reader 3, Median (IQR)		Reader 1, Median (IQR)	Reader 2, Median (IQR)	Reader 3, Median (IQR)	
Overall image quality	4(4–4)	4(4–4)	4(4–5)	0.729	3(3–4)	3(3–3)	3(3–3)	0.701	5(4–5)	5(4–5)	5(4–5)	0.797
Bladder wall delineation	4(4–4)	4(3.5–4.5)	4(4-4.5)	0.716	3.5(3–4)	3(3–4)	3.5(3–4)	0.649	5(4–5)	5(4.5-5)	5(4.5-5)	0.775
Ureterovesical junction delineation	4(3–4)	3(3–4)	4(3–4)	0.651	3(2–3)	3(3–4)	3(3–4)	0.645	4(4–4)	4(4–5)	4(4–5)	0.698
Breathing artifact	4(3–4)	3(3–4)	4(3–4)	0.788	5(4–5)	5(5–5)	5(5–5)	0.662	5(5–5)	5(5–5)	5(5–5)	0.853
Bowel motion artifact	3(3–3)	3(3–3)	3(3–3)	0.845	4(4–4)	4(4–5)	4(4–4)	0.657	5(5–5)	5(5–5)	5(5–5)	0.721
Urine flow artifact	5(4–5)	5(5–5)	5(5–5)	0.812	3(3–4)	4(3–4)	3(3–4)	0.795	4(4–4)	4(4–4)	4(3–4)	0.800
Diagnostic confidence	4(4–4)	4(4–5)	4(4–4)	0.709	3(3–3)	3.5(3–4)	4(3–4)	0.683	4(4–5)	4(4–5)	4(4–5)	0.754

ICC, intraclass correlation coefficient

**Table 5:** Bladder wall delineation based on four anatomical parts

	T2-TSE			ICC	HASTE			ICC	DL-HASTE			ICC
	Reader 1, Median (IQR)	Reader 2, Median (IQR)	Reader 3, Median (IQR)		Reader 1, Median (IQR)	Reader 2, Median (IQR)	Reader 3, Median (IQR)		Reader 1, Median (IQR)	Reader 2, Median (IQR)	Reader 3, Median (IQR)	
Bladder dome	3(3–4)	3(2–3)	3(2–4)	0.874	4(3–4)	4(3–4)	3(3–4)	0.659	5(4–5)	5(5–5)	5(4–5)	0.799
Bladder base	5(4–5)	5(4.5-5)	5(4–5)	0.671	3(3–3)	3(3–4)	3(3–4)	0.615	5(5–5)	5(5–5)	5(5–5)	0.705
Anterior bladder wall	4(4–4)	4(3-4.5)	4(3–5)	0.836	4(4–4)	4(3–4)	4(3–4)	0.732	5(5–5)	5(5–5)	5(5–5)	0.731
Posterior bladder wall	4(4–5)	4(3–5)	4(3-4.5)	0.766	3(3–4)	3(3–4)	4(3–4)	0.623	5(4–5)	5(5–5)	5(5–5)	0.821

**Table 6:** Statistical differences between T2-TSE, standard HASTE, DL-HASTE regarding qualitative metrics

	T2-TSE vs. DL-HASTE	HASTE vs. DL-HASTE	T2-TSE vs. HASTE
Overall image quality			
	< 0.001	< 0.001	< 0.001
Bladder wall delineation			
	< 0.001	< 0.001	< 0.001
Ureterovesical junction delineation			
	< 0.001	< 0.001	< 0.001
Breathing Artifact			
	< 0.001	< 0.05	< 0.001
Urine flow artifact			
	< 0.001	< 0.05	< 0.001
Bowel motion artifact			
	< 0.001	< 0.001	< 0.001
Diagnostic confidence			
	< 0.001	< 0.001	< 0.001

### Qualitative analysis

**Fig. 3:**
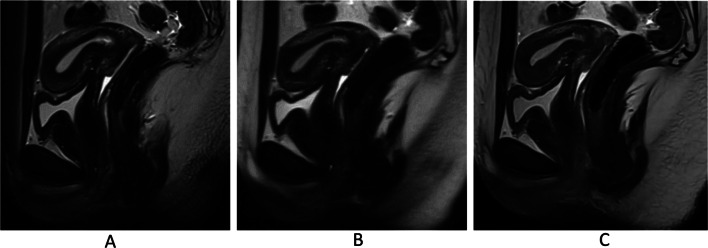
Overall image quality of T2-TSE (**A**), HASTE (**B**), and DL-HASTE (**C**). Image quality was rated excellent by all three readers on both T2-TSE and DL-HASTE. The image quality of HASTE was rated good by only one reader, while the other two rated it as moderate, primarily due to blurriness

Overall image quality was highest for DL-HASTE (average score: 5) followed by T2-TSE (average score: 4) and standard HASTE (average score: 3) (*P* < .001; Tables 4 and 6) (Figs. 2 and 3). Most T2-TSE images exhibited good image quality with minor movement artifacts and good bladder wall delineation. Only four cases (8%) of the T2-TSE datasets had excellent overall image quality without motion artifacts (Fig. 3A). No HASTE image had excellent image quality due to blurriness (Fig. 3B). DL-HASTE outperformed the other two sequences by effectively reducing the motion artifacts and blurriness (Fig. 3C).

**Fig. 4:**
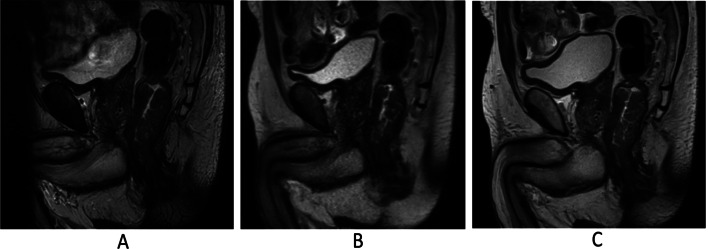
Delineation of the bladder wall on T2-TSE (**A**), HASTE (**B**), and DL-HASTE (**C**). The delineation of the bladder dome is severely affected by bowel motion artifact on T2-TSE (**A**), but clearly depicted by DL-HASTE (**C**)

**Fig. 5:**
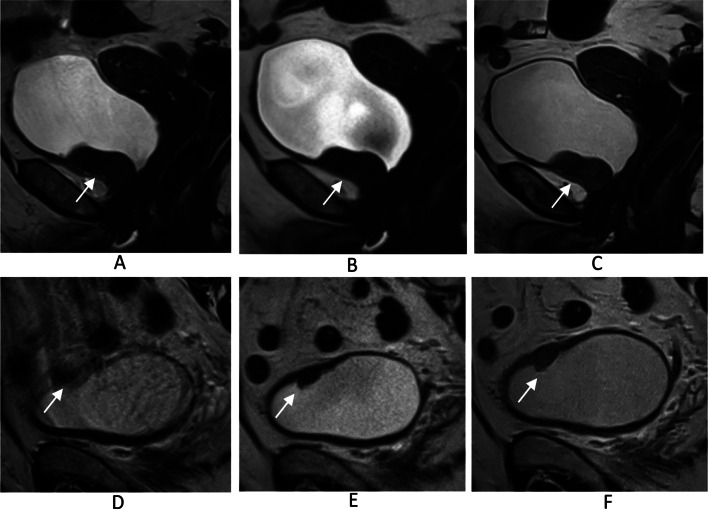
Two cases of bladder cancer (patient 1, **A**-**C**; patient 2, **D**-**F**). Patient 1 had bladder cancer (arrow) at the bladder base, which was best depicted on DL-HASTE (**C**). Patient 2 had bladder cancer at the bladder dome (arrow), which was also best depicted on DL-HASTE (**F**) but was severely blurred on T2-TSE (**D**)

DL-HASTE demonstrated significantly better delineation of bladder wall and ureterovesical junction compared to T2-TSE and HASTE (*P* < .001; Tables 4 and 6) (Fig. 4). The scores in Table 4 for bladder wall delineation represent averaged values for four anatomical parts of bladder wall (bladder dome, bladder base, anterior bladder wall and posterior bladder wall). Table 5 further illustrates the specific assessment based on separate anatomical parts of bladder wall in the same metric. Notably, the bladder dome, which can be severely affected by bowel motion artifacts, showed the highest difference in image quality ratings (Figs. 4 and 5).

**Fig. 6:**
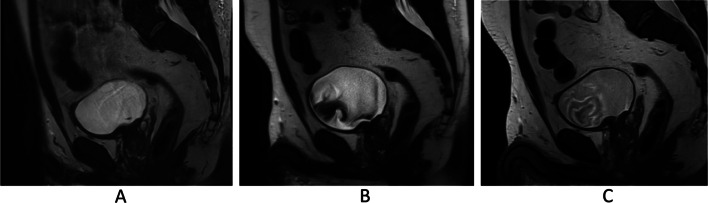
Urine flow artifacts on T2-TSE (**A**), HASTE (**B**), and DL-HASTE (**C**). Urine flow artifacts were evident on both HASTE (**B**) and DL-HASTE (**C**), whereas they were minimal on T2-TSE (**A**). In contrast, breathing and bowel motion artifacts were more pronounced on T2-TSE but absent on HASTE and DL-HASTE

Both HASTE and DL-HASTE effectively reduced artifacts from breathing and bowl motion compared to T2-TSE (*P* < .001). However, urine flow artifacts within the bladder lumen were frequently found on HASTE (average score: 3.3) and DL-HASTE (average score: 4) (*P* < .05), while these artifacts were mostly absent or minimal on T2-TSE (average score: 5) (*P* < .001) (Figs. 2 and 6), see Tables 4 and 6.

Diagnostic confidence in assessment of the bladder was good for T2-TSE and DL-HASTE (average score: 4, respectively), moderate for HASTE (average score: 3.5 3) (*P* < .001; Fig. 2; Table 6). By combining T2-TSE with DL-HASTE, all readers achieved the highest confidence (average score: 5; *P* < .001). In contrast, combining T2-TSE with HASTE resulted in a lower improvement of diagnostic confidence (average score: 4.5; *P* < .001).

### Quantitative analysis

**Fig. 7:**
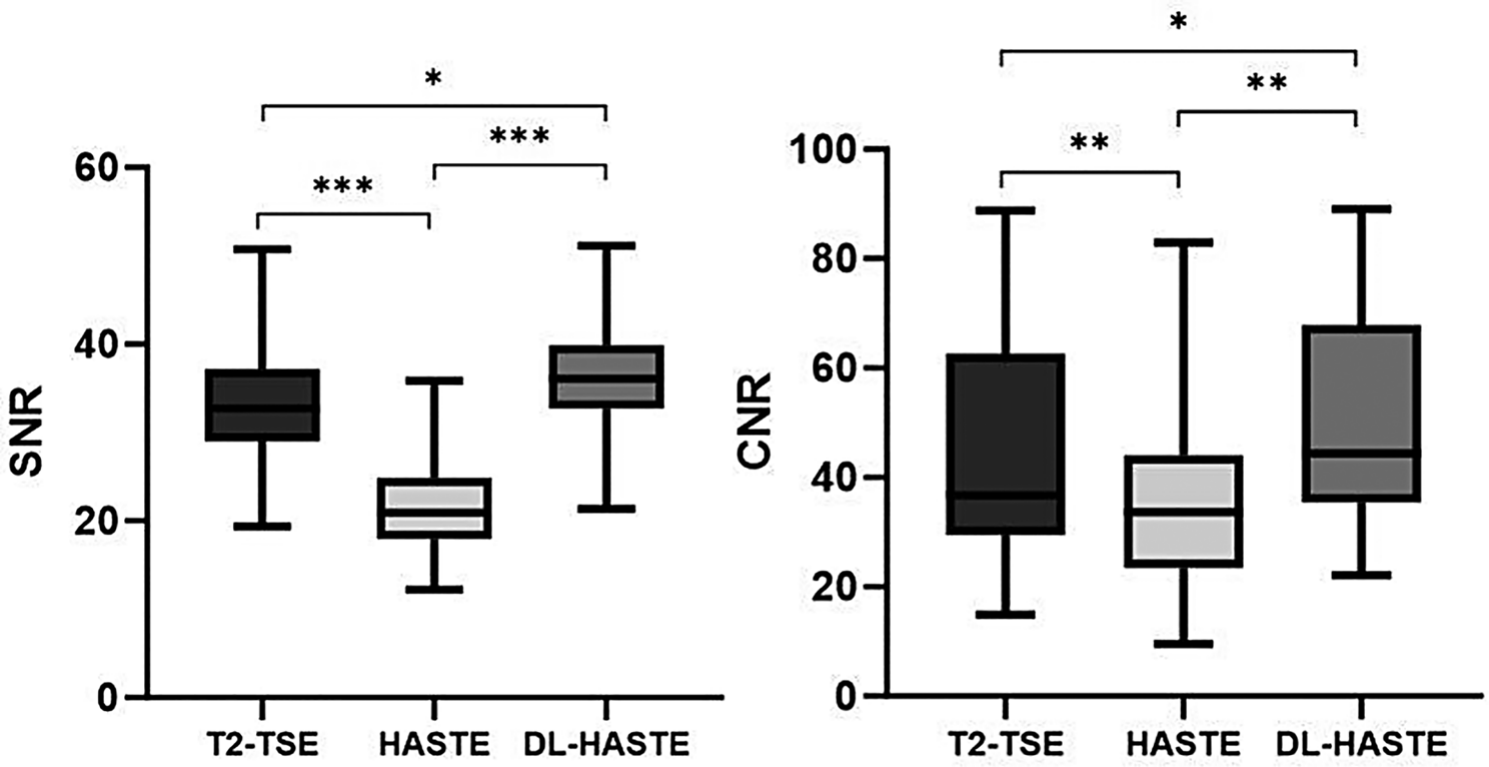
Box plots showed the differences in SNR and CNR among T2-TSE, standard HASTE and DL-HASTE, all the differences were statistically significant (*, *P* < .05; **, *P* < .01; ***, *P* < .001)

The quantitative results are presented in Fig. 7. The values of fat-SNR (36.3 ± 6.3 [SD]) and muscle-fat CNR (50.3 ± 19.7 [SD]) were the highest for DL-HASTE followed by T2-TSE (33.1 ± 6.3 [SD]; *P* =.02 and 44.3 ± 21.0 [SD]; *P* =.02, respectively) and HASTE (21.7 ± 5.4 [SD]; *P* < .001 and 35.8 ± 17.5 [SD]; *P* =.001, respectively).

## Discussion

This prospective study investigated the feasibility of ultrafast sagittal 3 mm DL-HASTE for urinary bladder imaging. DL-HASTE outperformed both T2-TSE and HASTE by effectively suppressing motion artifacts compared to T2-TSE and enhancing image sharpness compared to HASTE. The highest diagnostic confidence in urinary bladder evaluation was achieved with the combined use of T2-TSE and DL-HASTE.

Conventional T2-TSE imaging plays a crucial role in urinary bladder evaluation, as it provides excellent soft-tissue contrast between bladder wall, urine, and bladder tumors [1, 25]. However, inherent physiologic motion artifacts can compromise image quality, obscuring both anatomic and pathological details [6, 26], this is especially problematic for the sagittal imaging plane. Our study highlighted the limitations of the T2-TSE sequence: prolonged acquisition time introduced motion artifacts from breathing and bowel movements, leading to blurred bladder wall delineation and decreased SNR. While antiperistaltic agents can mitigate bowel motion artifacts in T2-TSE imaging and are recommended in urogenital MR imaging [27], their application warrants careful consideration due to potential precautions and complications [28–29]. Moreover, depending on pelvic region involvement and utilization of fast imaging protocols, some literature suggests the possibility of omitting antiperistaltic agents [30–31], enhancing patient comfort and streamlining clinical imaging workflow. Our findings demonstrate that integrating an ultrafast sagittal DL-HASTE sequence into the MRI protocol enables confident urinary bladder evaluation, even amidst motion artifacts encountered in T2-TSE imaging.

Standard HASTE sequences are widely used in clinical routine for fast T2w-imaging, especially in abdominal imaging [10–11, 20, 22, 32]. HASTE effectively reduces motion artifacts by allowing shorter acquisition time. However, compared with T2-TSE, HASTE suffers from lower SNR due to its longer echo train length [12–13]. In our study, inadequate image quality was notably apparent in the bladder base region, with limited distinction from adjacent structures such as the prostate and pelvic floor. Moreover, standard HASTE was susceptible to urine flow artifacts, potentially leading to misinterpretation as intraluminal bladder pathologies. Overall, standard HASTE presents significant limitations in urinary bladder imaging and is primarily utilized in clinical settings as an initial MR sequence for general pelvic assessment.

DL reconstruction presents an innovative approach to enhancing MR imaging quality while drastically reducing scanning time. Utilizing a variational network, DL reconstruction generates high-quality images from undersampled k-space or noisy image data [15, 16, 17, 18]. Ren et al. used DL-accelerated T2-TSE in female pelvic MRI, demonstrating a notable enhancement in image quality [15]. However, the acquisition time of the DL-T2-TSE sequence was almost 2 min, leaving the sequence susceptible to motion artifacts. In contrast, our DL-accelerated HASTE sequence facilitated scanning of the entire pelvis in under 30 s. To mitigate specific absorption rate (SAR) limitations at 3 Tesla, we employed a DL-HASTE sequence with a variable flip angle (FA) as previously described [20]. With the application of DL-HASTE, bladder wall delineation significantly improved, particularly in the bladder dome and ventral bladder walls. These regions are crucial as they may be affected by various pathological conditions, including bladder cancer, bladder anomalies (notably urachus anomalies with associated inflammatory and malignant changes), and pathologies from adjacent organs such as the sigmoid colon (including inflammatory involvement and tumor infiltration). In comparison to standard HASTE, we observed milder urine flow artifacts with DL-HASTE, likely attributed to its rapid acquisition scheme. But for now, the presence of these artifacts precludes the standalone use of DL-HASTE as an MR sequence for T2-weighted imaging of the urinary bladder. However, DL-HASTE is a promising additional MR sequence to T2-TSE, serving not only as a rapid initial MR sequence for general pelvic assessment, but also providing high diagnostic value in evaluating the urinary bladder wall.

Our study has some limitations: This study was exploratory in nature and sample size was determined pragmatically based on anticipated recruitment. While sufficient for feasibility assessment and image quality comparison, larger cohorts will be needed in future studies to validate these findings, particularly for specific diagnostic subgroups to make our conclusion more convincing and generalizable. Our study used only DL techniques in the sagittal plane. We believe this approach is both feasible and comprehensive for clinical evaluation in bladder imaging as a supplementary diagnostic sequence in pelvic imaging because most artifacts are typically encountered in this plane. Hence, DL-HASTE improves image quality of the bladder dome, which is most susceptible to motion artifacts in the sagittal orientation and cannot be visualized directly in the axial orientation.

In conclusion, DL-HASTE allows for ultrafast imaging of the bladder with robust, high image quality and is a useful addition to the standard MRI workup of the pelvis.

## Data Availability

The data is available from the autors upon reasonable request.

## Abbreviations

HASTE: Half-Fourier single-shot turbo spin echo sequence
DL: Deep learning
FA: Flip angle
TSE: Turbo spin echo sequence
SD: Standard deviation
SI: Signal intensity
CNR: Contrast-to-noise-ratio
SNR: Signal-to-noise-ratio
ROI: Region of interest
MRI: Magnetic resonance imaging
